# Association of Coming Out as Lesbian, Gay, and Bisexual+ and Risk of Cigarette Smoking in a Nationally Representative Sample of Youth and Young Adults

**DOI:** 10.1001/jamapediatrics.2020.3565

**Published:** 2020-10-26

**Authors:** Alyssa F. Harlow, Dielle Lundberg, Julia R. Raifman, Andy S. L. Tan, Carl G. Streed, Emelia J. Benjamin, Andrew C. Stokes

**Affiliations:** 1 Department of Epidemiology, Boston University School of Public Health, Boston, Massachusetts; 2 Department of Global Health, Boston University School of Public Health, Boston, Massachusetts; 3 Department of Health Law, Policy and Management, Boston University School of Public Health, Boston, Massachusetts; 4 Annenberg School for Communication, University of Pennsylvania, Philadelphia; 5 Section of General Internal Medicine, Department of Medicine, Boston University School of Medicine, Boston, Massachusetts; 6 Department of Medicine, Boston University School of Medicine, Boston, Massachusetts

## Abstract

**Importance:**

Coming out as lesbian, gay, bisexual, or other identities besides heterosexual (LGB+) may represent a susceptible period for cigarette smoking initiation in youth and young adults.

**Objective:**

To assess whether young people who change their sexual identity have higher risk of cigarette smoking initiation and current smoking compared with those with consistent sexual identities.

**Design, Setting, and Participants:**

This cohort study used data from the nationally representative Population Assessment of Tobacco and Health study (wave 1, 2013-2014; wave 2, 2014-2015; wave 3, 2015-2016; wave 4, 2016-2018). Youth and young adults aged 14 to 29 years who were never smokers at wave 1 were included in this study. Analysis began October 2018 and ended June 2020.

**Exposures:**

Consistent sexual identity (consistently heterosexual, consistently LGB+) vs changing sexual identity (coming out as LGB+, other LGB+ patterns) based on 4 waves of sexual identity data. Identities were further classified by distinguishing between bisexual and lesbian, gay, and other nonheterosexual identities.

**Main Outcomes and Measures:**

Smoking initiation and current cigarette smoking at wave 4.

**Results:**

Among 7843 individuals who never smoked at wave 1, 6991 (90.7%) reported a consistent sexual identity, and 852 (9.3%) changed sexual identity across waves. The mean (SE) baseline age of participants who reported consistent heterosexuality was 20.1 (0.8) years; consistently LGB+, 20.0 (3.7) years; coming out as LGB+, 18.0 (2.9) years, and other LGB+ pattern, 20.3 (3.8) years. A total of 14.1% (weighted) initiated smoking, and 6.3% were current smokers at wave 4. Compared with consistently heterosexual identities, coming out as LGB+ (23% vs 13%; odds ratio [OR], 1.72; 95% CI, 1.34-2.20), consistently LGB+ identities (17% vs 13%; OR, 1.45; 95% CI, 1.03-2.04), and other LGB+ patterns (17% vs 13%; OR, 1.47; 95% CI, 1.04-2.08) were positively associated with smoking initiation by wave 4. Compared with consistently heterosexual identities, ORs for smoking initiation were 2.24 (28% vs 13%; 95% CI, 1.72-2.92) for coming out as bisexual, 1.99 (23% vs 13%; 95% CI, 1.20-3.29) for consistently LGB+ with change to/from bisexual, and 2.20 (23% vs 13%; 95% CI, 1.40-3.46) for other LGB+ patterns with change to/from bisexual identity. Current smoking estimates were similar to those for smoking initiation.

**Conclusions and Relevance:**

Compared with consistently heterosexual identities, changing sexual identity over follow-up was associated with smoking initiation and current smoking. The risk associated with changing sexual identities was concentrated among participants coming out as bisexual or reporting other changes in their identity to/from being bisexual. More research is needed on mechanisms underlying the association between changing sexual identity and smoking initiation to inform tailored prevention programs and tobacco regulations.

## Key Points

**Question:**

Is changing sexual identity associated with increased risk of cigarette smoking initiation and current smoking?

**Findings:**

In this nationally representative cohort study of 7843 youth and young adults, those who changed their sexual identity from heterosexual to bisexual were more likely than those with consistent heterosexual identities to initiate smoking and be current smokers during 3 years of follow-up. There was no increased risk for youth and young adults changing from heterosexual to lesbian, gay, or other nonheterosexual identities.

**Meaning:**

In this study, coming out as bisexual was associated with increased risk of initiating cigarette smoking and current smoking in youth and young adults.

## Teaser

This cohort study assesses whether young people who change their sexual identity are associated with having higher risk of cigarette smoking initiation and current smoking compared with those with consistent sexual identities.

## Introduction

Significant disparities in tobacco use exist for lesbian, gay, bisexual, and other nonheterosexual identity (LGB+) cisgender populations compared with heterosexual populations.^1,2^ LGB+ young people have higher rates of cigarette smoking, smoking cigarettes at earlier ages, and smoking with more intensity than heterosexual youth.^3,4,5,6,7^ The prevalence of smoking among LGB+ populations is approximately 22%, compared with 14% among heterosexual populations.^8^ Within LGB+ groups, bisexual populations have the highest prevalence of current smoking, at more than 30%.^8^ Cigarette smoking is associated with 2 to 4 times the risk of coronary heart disease and stroke,^9^ with 25 times the risk of lung cancer,^9^ and with a myriad of other health consequences,^9^ putting LGB+ youth at increased risk of smoking-related disease. Addressing LGB+ disparities in cigarette smoking is a public health priority.^10,11^

Most research on LGB+ smoking disparities considers sexual identity a static phenonemenon.^12^ However, establishing one’s sexual identity, often referred to as *coming out*, is a multistage developmental process.^13^ Sexual identity development can differ from person to person,^14^ sexual identity can change over time among individuals, and the stage or timing of one’s sexual identity development may play an important role in risk behaviors.^15,16,17,18,19^ LGB+ young people just beginning a coming out process may be more at risk of cigarette smoking than those who have consistently identified as LGB+ for some time. Establishing a new identity may result in more internalized and externalized stress as a result of discrimination, social exclusion, experiences of identity rejection, or internalized homophobia, leading to smoking as a coping mechanism.^20^ Young people with a new LGB+ identity also may be exposed to new social environments with more positive tobacco-related social norms^21,22^ or encounter LGB+-targeted tobacco marketing.^23^ Sexual identity is complex, and different stressors exist among individuals coming out to or from different identities. For example, the tobacco risk of a heterosexual person coming out as bisexual may differ from someone coming out as gay or lesbian and may also differ from a gay or lesbian person coming out as bisexual.^1^

Previous research has been limited in its ability to explore nuances of sexual identity changes in relation to smoking behaviors because of a lack of longitudinal data that measure sexual identity. The Population Assessment of Tobacco and Health (PATH) study provides an opportunity to examine changes in sexual identity and cigarette smoking on a national level, as the nationally representative survey asks youth about sexual identity and smoking behaviors over multiple waves of data collection. The present study aimed to determine whether youth and young adults who report a change in sexual identity have higher risk of cigarette smoking than young people with consistent sexual identities over follow-up using 4 waves of data from the PATH study (2013-2018). We hypothesize that youth and young adults who come out as LGB+ or report other changes in sexual identity during the follow-up period have higher risk of initiating cigarette smoking and current smoking than those with consistent heterosexual identities. Because prior studies reported that bisexual populations have higher rates of smoking compared with other lesbian, gay, and other nonheterosexual (LG+) populations,^24,25^ we also hypothesize that the risk of smoking initiation and current smoking is greater for young people who come out as bisexual than for those who come out as LG+.

## Methods

### Study Sample

We used 4 waves of data from the PATH study, established in 2013 by the National Institutes of Health and the US Food and Drug Administration to study tobacco product use and health. PATH sampled more than 49 000 youth and adults in the United States using a 4-stage stratified area probability sample design.^26^ Participants completed 4 waves of data approximately 12 months apart. We used deidentified data from the adult and youth restricted use files from wave 1 (September 2013-December 2014), wave 2 (October 2014-October 2015), wave 3 (October 2015-October 2016), and wave 4 (December 2016-January 2018). Output of unweighted frequencies were not allowed from the restricted use files. We restricted our analysis to participants aged 14 to 29 years who were never cigarette smokers at wave 1 and did not identify as transgender by wave 4 (eFigure in the Supplement). This study relies on deidentified data and was deemed exempt by the Boston University Medical Center Institutional Review Board.

### Outcome: Cigarette Smoking Initiation

Our primary outcome was cigarette smoking initiation, defined as ever smoking a cigarette by wave 4 and measured with the question, “Have you ever smoked a cigarette, even 1 or 2 puffs?” We also examined current smoking at wave 4, defined as past 30-day use and measured with the question, “In the past 30-days, have you smoked a cigarette, even 1 or 2 puffs?” Past 30-day smoking is a standard measure for current smoking among youth and is associated with smoking in adulthood.^27,28^

### Sexual Identity Categories

We distinguished between consistent sexual identities and changing identities over the 3-year follow-up. At each wave, participants were asked the following question on sexual identity: “Do you consider yourself to be…(1) straight, (2) lesbian or gay, (3) bisexual, (4) something else?” We dichotomously classified participants as either heterosexual or LGB+ at each wave. LGB+ identities included lesbian/gay, bisexual, or something else (other than heterosexual). The something else category may include other identities such as queer/asexual, etc, or those who do not use labels or are questioning their identity.^26^ We then further categorized participants by whether they had a consistent identity across waves or if they came out during follow-up. We defined coming out as changing one’s reported sexual identity from heterosexual to LGB+ over follow-up. Consistent identities included participants who were consistently heterosexual or consistently LGB+ at all 4 waves. Coming out identities included participants who identified as heterosexual at wave 1 and identified as LGB+ by wave 4. Approximately 4% of the sample reported other identity patterns (eg, changed from LGB+ to heterosexual). Our primary independent variable was a 4-category mutually exclusive sexual identity variable: (1) consistently heterosexual, (2) consistently LGB+, (3) coming out LGB+, and (4) other LGB+ patterns.

As prior studies indicate high smoking prevalence among bisexual youth,^24,25^ we classified participants by whether they identified as bisexual at any point over follow-up for an additional independent variable (Table 1). We divided our original 4-level variable into an 8-level variable accounting for bisexual identity: (1) consistently heterosexual, (2) consistently LG+, (3) consistently bisexual, (4) consistently LGB+ with change to/from bisexual, (5) coming out bisexual, (6) coming out LG+, (7) other LGB+ patterns with change to/from bisexual, and (8) other LG+ patterns. Table 1 provides definitions for the 8 identity categories.

**Table 1.** Eight-Level Sexual Identity Categories Over 4 Waves of the Population Assessment of Tobacco and Health Study, 2013-2018

Sexual identity category	Individuals, No. (%)	Derived from original identity category^a^	Description
Consistently heterosexual	6707 (85.5)	Consistently Heterosexual	Participants who do not identify as lesbian, gay, bisexual, or other nonheterosexual identities over the entire 3-y follow-up
Consistently LG+	100 (12.8)	Consistently LGB+	Participants who consistently identify as lesbian, gay, or other nonheterosexual identities over the 3-y follow-up; excluding bisexual identity
Consistently bisexual	85 (1.1)	Consistently LGB+	Participants who identify as bisexual over the entire 3-y follow-up
Consistently LGB+ with change to/from bisexual	99 (1.3)	Consistently LGB+	Participants who identify as lesbian, gay, bisexual, or other nonheterosexual identities over the 3-y follow-up but change between a lesbian/gay/other nonheterosexual identity and a bisexual identity; these include participants who change from bisexual to lesbian/gay/other identities and vice versa
Coming out bisexual	339 (4.3)	Coming out LGB+	Participants who identify as heterosexual at wave 1 and change to a bisexual identity over follow-up; this may also include participants who identify as lesbian/gay/other nonheterosexual identity in addition to bisexual over follow-up
Coming out LG+	163 (2.1)	Coming out LGB+	Participants who identify as heterosexual at wave 1 and change to a lesbian, gay, or other non-heterosexual identity over follow-up. These participants do not identify as bisexual at any point.
Other LGB+ patterns with change to/from bisexual	189 (2.4)	Other LGB+ patterns	Participants who change between a bisexual and heterosexual identity over follow-up and do not meet the definition for coming out bisexual; this may also include participants who identify as lesbian/gay/other nonheterosexual identity in addition to bisexual and heterosexual over follow-up
Other LG+ patterns	161 (2.1)	Other LGB+ patterns	Participants who change between a lesbian/gay/other nonheterosexual identity and heterosexual identity over follow-up and do not meet the definition for coming out LG+; these participants do not identify as bisexual at any point

Abbreviations: LG+, lesbian, gay, and other nonheterosexual identities; LGB+, lesbian, gay, bisexual, and other nonheterosexual identities.

^a^ Eight-level bisexual identities were derived from the 4-level identity variable. Consistently heterosexual participants identified as heterosexual at all 4 waves; consistently LGB+ participants identified as LGB+ at all 4 waves; coming out LGB+ identified as heterosexual at wave 1 and LGB+ by wave 4; Participants with other LGB+ patterns changed sexual identity over follow-up but did not meet the definition of coming out LGB+.

### Assessment of Covariates

At wave 1, participants reported their age (continuous), sex (male, female), race/ethnicity (non-Hispanic Black, non-Hispanic White, non-Hispanic Asian or other race, Hispanic), urban residence (urban, nonurban), and census region (Northeast, South, Midwest, West). Education was measured for participants 18 years and older, and parental education was measured for participants younger than 18 years (<high school or GED, high school graduate, >high school). Parental education was the only measure of socioeconomic status in the wave 1 youth survey.

### Statistical Analysis

We compared wave 1 characteristics across each sexual identity category and tested the association between sexual identities and our 2 outcomes (smoking initiation and current smoking at wave 4) using multivariable logistic regression. We separately modeled each outcome as a function of identity categories, with consistently heterosexual as the reference, adjusting for covariates associated with cigarette smoking and sexual identity, and temporally preceding sexual identity changes. We did not adjust for potential mediators, including other substance use or mental health disorders. Final models adjusted for age, sex, race/ethnicity, education, urban residence, and census region. We calculated adjusted predicted probabilities of wave 4 cigarette smoking assuming the mean value of model covariates.^29^ We repeated the analysis using the 8-level identity measure accounting for bisexual identity.

We stratified our primary analysis by sex and age at baseline (<18 vs ≥18 years) and included interaction terms between sexual identity and sex as well as sexual identity and age. We compared attrition between wave 1 LGB+ and heterosexual participants and compared baseline characteristics of those retained vs lost to follow-up. Missing data on exposure, outcome, and covariates (n = 462) were assumed to be missing at random and imputed using multiple imputation with 5 data sets. For all estimates, we used population weights and replicate weights using the balanced repeated replication method with the Fay adjustment (ρ = 0.3) for inferences generalizable to the US population.^26^ Analyses were conducted using SAS version 9.4 (SAS Institute). Analysis began October 2018 and ended June 2020.

## Results

Our study included 7843 participants aged 14 to 29 years who were never cigarette smokers at wave 1. Table 2 and eTable 1 in the Supplement present baseline characteristics stratified by our 4-level and 8-level identity categories, respectively. Most participants (6707 [87.1%]) reported a consistently heterosexual identity across waves, 284 (3.6%) reported a consistently LGB+ identity, 502 (5.0%) came out as LGB+ over follow-up, and 350 (4.4%) reported other LGB+ patterns (Table 2). Among other LGB+ patterns, 62% (weighted) changed from LGB+ at wave 1 to heterosexual over follow-up (eTable 2 in the Supplement). The mean (SE) baseline age of participants who reported consistent heterosexuality was 20.1 (0.8) years; consistently LGB+, 20.0 (3.7) years; coming out as LGB+, 18.0 (2.9) years; and other LGB+ pattern, 20.3 (3.8) years. Attrition was similar for wave 1 LGB+ and heterosexual participants (31.1% vs 30.5%). There were no substantive differences in baseline characteristics between those retained and lost to follow-up (eTable 3 in the Supplement).

**Table 2.** Descriptive Baseline Characteristics Stratified by 4-Level Sexual Identity Categories Among 7843 Individuals Who Never Smoked Cigarettes From the Population Assessment of Tobacco and Health Study, 2013-2018

Wave 1 characteristic^a^	Consistently heterosexual^b^	Consistently LGB+^b^	Coming out LGB+^b^	Other LGB+ pattern^b^
Total individuals, No. (%)	6707 (87.1)	284 (3.6)	502 (5.0)	350 (4.4)
Age, mean (SE), y	20.1 (0.8)	20.0 (3.7)	18.0 (2.9)	20.3 (3.8)
Female, %	50.7	64.4	62.6	63.5
Race/ethnicity, %				
Non-Hispanic White	52.9	53.4	53.4	39.1
Non-Hispanic Black	14.9	16.6	13.3	19.6
Hispanic	20.3	16.1	18.2	30.5
Asian or other race^c^	11.8	13.8	15.0	10.8
Education, %^d^				
<High school	11.0	12.6	12.3	25.5
Graduated high school	24.6	22.3	24.6	25.2
>High school	64.5	65.1	63.1	49.3
US census region, %				
Northeast	17.8	17.1	12.4	18.2
Midwest	21.1	23.8	29.0	21.1
South	36.2	33.5	34.8	38.3
West	24.9	25.5	23.8	22.4
Urban residing, %	95.5	96.7	95.5	97.3

Abbreviation: LGB+, lesbian, gay, bisexual, and other nonheterosexual identities.

^a^ Descriptive statistics calculated using sample-weighted percentages and sample-weighted means.

^b^ Consistently heterosexual participants identified as heterosexual at all 4 waves; consistently LBG+ participants identified as LGB+ at all 4 waves; and coming out LGB+ identified as heterosexual at wave 1 and LGB+ by wave 4. Participants with other LGB+ patterns changed sexual identity over follow-up but did not meet the definition of coming out LGB+.

^c^ Other races included individuals reporting more than 1 race, American Indian/Alaska Native, and Native Hawaiian/Pacific Islander.

^d^ For participants younger than 18 years, parental education is reported.

### Smoking Initiation by Wave 4

By wave 4, 14.1% of participants reported ever cigarette smoking. Adjusted predicted probabilities of ever smoking were 23.8% (95% CI, 19.3%-28.2%) for coming out LGB+, 21.1% (95% CI, 15.3%-26.9%) for other LGB+ patterns, 20.9% (95% CI, 15.3%-26.5%) for consistently LGB+, and 15.4% (95% CI, 13.8%-16.9%) for consistently heterosexual participants. Coming out as LGB+ was associated with 72% greater adjusted odds of smoking initiation compared with consistently heterosexual identities (odds ratio [OR], 1.72; 95% CI, 1.34-2.20) (Table 3). Participants who reported other LGB+ patterns were 1.47 (95% CI, 1.04-2.08) times more likely and consistently LGB+ participants were 1.45 (95% CI, 1.03-2.04) times more likely than consistently heterosexual participants to report smoking initiation.

**Table 3.** Cigarette Smoking at Wave 4 by 4-Level Sexual Identity Categories Among 7843 Individuals Who Never Smoked Cigarettes at Wave 1 From the Population Assessment of Tobacco and Health Study, 2013-2018

Sexual identity category	Cigarette use, %^a^	Odds ratio (95% CI)	Adjusted odds ratio (95% CI)^b^	Predicted probabilities (95% CI)^b^^,^^c^
**Smoking initiation by wave 4**^**d**^				
Total	14.1	NA	NA	NA
Consistently heterosexual	13.3	1 [Reference]	1 [Reference]	0.154 (0.138-0.169)
Consistently LGB+	16.9	1.33 (0.94-1.88)	1.45 (1.03-2.04)	0.209 (0.153-0.265)
Coming out LGB+	23.0	1.98 (1.51-2.60)	1.72 (1.34-2.20)	0.234 (0.193-0.282)
Other LGB+ patterns	17.3	1.39 (0.97-2.01)	1.47 (1.04-2.08)	0.211 (0.153-0.269)
**Current smoker at wave 4**^**e**^				
Total	6.3	NA	NA	NA
Consistently heterosexual	5.8	1 [Reference]	1 [Reference]	0.069 (0.060-0.077)
Consistently LGB+	8.3	1.49 (0.90-2.45)	1.63 (1.00-2.66)	0.107 (0.060-0.154)
Coming out LGB+	11.5	2.12 (1.55-2.89)	1.78 (1.33-2.39)	0.116 (0.085-0.147)
Other LGB+ patterns	8.5	1.54 (0.98-2.44)	1.63 (1.03-2.56)	0.107 (0.063-0.151)

Abbreviations: LGB+, lesbian, gay, bisexual, and other nonheterosexual identities; NA, not applicable.

^a^ Sample-weighted percentages.

^b^ Regression models and predicted probabilities were sample weighted and adjusted for urban residence, sex, race/ethnicity, education, and census region.

^c^ Predicted probabilities calculated at the mean of model covariates.

^d^ Reported ever smoking by wave 4.

^e^ Defined as past 30-day smoking at wave 4.

Participants who came out as bisexual had 2.24 (95% CI, 1.72-2.92) times the adjusted odds of smoking initiation by wave 4 compared with consistently heterosexual young people (Table 4). Participants with other LGB+ patterns that included change to/from bisexuality had an adjusted OR of 2.20 (95% CI, 1.40-3.46). Participants with consistently LGB+ identities that included change to/from bisexuality had an adjusted OR of 1.99 (95% CI, 1.20-3.29). There was no association for participants who came out as LG+ (OR, 0.91; 95% CI, 0.52-1.60), those with consistently LG+ identities (OR, 0.90; 95% CI, 0.43-1.87) or those with other LG+ patterns (OR, 0.91; 95% CI, 0.55-1.49). Estimates for consistently bisexual participants were imprecise but indicated an association with elevated risk (OR, 1.60; 95% CI, 0.89-2.88). The Figure presents predicted probabilities by the 8-level sexual identity categories.

**Table 4.** Cigarette Smoking at Wave 4 by 8-Level Identity Categories Among 7843 Individuals Who Never Smoked Cigarettes at Wave 1 From the Population Assessment of Tobacco and Health Study, 2013-2018

Sexual identity category	Cigarette use, %^a^	Odds ratio (95% CI)	Adjusted odds ratio (95% CI)^b^	Predicted probabilities (95% CI)^b^^,^^c^
**Smoking initiation by wave 4**^**d**^				
Total	14.1	NA	NA	NA
Consistently heterosexual	13.3	1 [Reference]	1 [Reference]	0.153 (0.138-0.169)
Consistently LGB+ identities				
Consistently LG+	11.5	0.80 (0.38-1.67)	0.90 (0.43-1.87)	0.141 (0.054-0.228)
Consistently bisexual	17.1	1.36 (0.72-2.58)	1.60 (0.89-2.88)	0.225 (0.123-0.327)
Consistently LGB+ with change to/from bisexual	22.6	2.02 (1.16-3.53)	1.99 (1.20-3.29)	0.264 (0.164-0.365)
Coming out LGB+ identities				
Coming out bisexual	28.3	2.59 (1.94-3.47)	2.24 (1.72-2.92)	0.289 (0.235-0.342)
Coming out LG+	13.5	1.03 (0.58-1.84)	0.91 (0.52-1.60)	0.141 (0.073-0.210)
Other LGB+ patterns				
Other LGB+ patterns with change to/from bisexual	23.4	2.12 (1.30-3.46)	2.20 (1.40-3.46)	0.285 (0.194-0.377)
Other LG+ patterns	11.5	0.85 (0.50-1.45)	0.91 (0.55-1.49)	0.141 (0.079-0.202)
**Current smoker at wave 4**^**e**^				
Total	6.3	NA	NA	NA
Consistently heterosexual	5.8	1 [Reference]	1 [Reference]	0.068 (0.060-0.077)
Consistently LGB+ identities				
Consistently LG+	3.8	0.60 (0.20-1.82)	0.69 (0.22-2.14)	0.048 (0.00-0.100)
Consistently bisexual	8.2	1.47 (0.62-3.46)	1.77 (0.76-4.10)	0.115 (0.031-0.199)
Consistently LGB+ with change to/from bisexual	13.2	2.64 (1.34-5.20)	2.58 (1.36-4.89)	0.159 (0.071-0.248)
Coming out LGB+ identities				
Coming out bisexual	14.2	2.73 (1.96-3.80)	2.28 (1.64-3.15)	0.143 (0.103-0.183)
Coming out LG+	6.4	1.07 (0.57-2.00)	0.93 (0.51-1.69)	0.064 (0.027-0.100)
Other LGB+ patterns				
Other LGB+ patterns with change to/from Bisexual	10.6	2.04 (1.09-3.83)	2.07 (1.11-3.88)	0.132 (0.063-0.201)
Other LG+ patterns	6.5	1.13 (0.58-2.19)	1.23 (0.65-2.32)	0.083 (0.032-0.135)

Abbreviations: LG+, lesbian, gay, and other nonheterosexual identities; LGB+, lesbian, gay, bisexual, and something else besides heterosexual; NA, not applicable.

^a^ Sample-weighted percentages.

^b^ Regression models and predicted probabilities were sample weighted and adjusted for urban residence, sex, race/ethnicity, education, census region.

^c^ Predicted probabilities calculated at the mean of model covariates.

^d^ Reported ever smoking by wave 4.

^e^ Defined as past 30-day smoking at wave 4.

**Figure.**
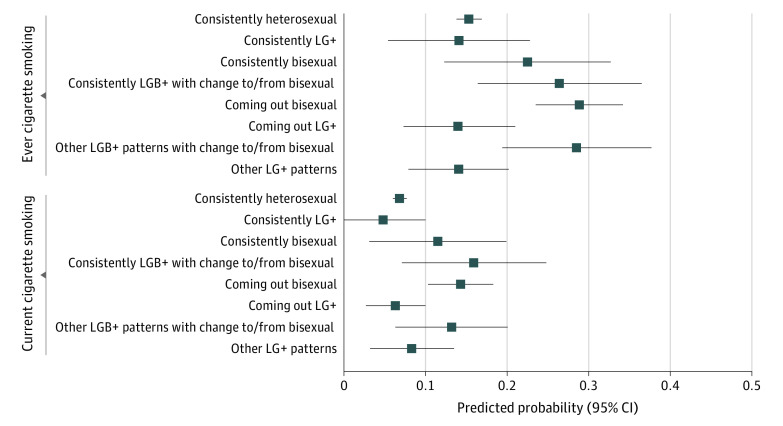
Adjusted Predicted Probability of Cigarette Smoking at Wave 4 by Sexual Identity Transitions From the Population Assessment of Tobacco and Health, 2013-2018 LG+ indicates lesbian, gay, and other nonheterosexual identities; LGB+, lesbian, gay, bisexual, and other nonheterosexual identities.

### Current Smoking at Wave 4

By wave 4, 6.3% of participants reported current smoking (Table 3). Adjusted ORs for current smoking were 1.78 (95% CI, 1.33-2.39) for coming out LGB+, 1.63 (95% CI, 1.03-2.56) for other LGB+ patterns, and 1.63 (95% CI, 1.00-2.66) for consistently LGB+ compared with consistently heterosexual participants (Table 3). When accounting for bisexual identity, all identities with change to or from bisexuality had approximately twice the odds of current smoking compared with consistently heterosexual identity (Table 4). Odds ratios for coming out LG+ and other LG+ patterns showed no association. Estimates for consistently LG+ identities indicated a reduced risk, but confidence intervals were wide and included no association.

### Sensitivity Analyses

Stratified estimates were less precise than primary models, but we identified some differences in smoking initiation by sex. Among male individuals, consistent LGB+ identities and other LGB+ patterns were not associated with increased risk of smoking initiation, although a positive association remained for coming out LGB+ (eTable 4 in the Supplement). There were no substantive differences by age (eTable 5 in the Supplement).

## Discussion

In this longitudinal nationally representative study, we found that participants who came out as bisexual or reported changes in their identity to/from being bisexual were twice as likely to initiate smoking and to be current smokers compared with consistently heterosexual participants. Participants who came out as LG+ did not have increased risk of smoking initiation or current smoking compared with consistently heterosexual participants.

Our findings provide evidence that changing sexual identities may be an important mechanism behind the LGB+ smoking disparity, particularly for bisexual young people. Results align with previous research on sexual identity fluidity and substance use risk^30^ and provide evidence that the association between coming out and smoking is present in a nationally representative sample. Our data are also supported by prior studies showing life event transitions (eg, losing a job, change in romantic partners, entering college) increase risk of smoking^31,32^ and other substance use.^33,34^ Our work demonstrates the timing and type of sexual identity development matters with regard to smoking behaviors in young people. Public health professionals should recognize the nuances of sexual identity and its effect on health behaviors,^35^ rather than homogenizing young people into binary and static sexual identities.^36^ This is particularly important given that in our nationally representative sample of youth and young adults, a majority of LGB+ participants reported a change in sexual identity over the 3-year follow-up.

Importantly, our results indicate that the smoking risk associated with changing sexual identities was entirely attributed to coming out as bisexual or reporting other changes in identity to/from being bisexual, rather than coming out as LG+. The elevated smoking risk was present for those changing between heterosexual and bisexual identities and for those changing between LG+ and bisexual identities. In addition, after accounting for bisexual identity, the association for consistently LG+ participants completely attenuated, while consistently bisexual participants had elevated smoking risk. Previous research shows bisexual young people have higher prevalence of smoking^8,25^ and other substance use^24^ compared with other LG+ groups. Prior data and our own findings highlight the importance of differentiating between bisexual and other LG+ identities in health research. Results also reveal the importance of studying changes between LGB+ identities in addition to changes between heterosexual and LGB+ identities. Categorizing participants into 1 homogenous sexual minority status group can obscure important disparities within the LGB+ population.

Young people changing sexual identities may develop smoking habits as a form of integration with new social environments.^21,22,24,37^ Smoking may be used to establish oneself in a new community or to reduce social anxiety associated with new social groups.^24,38^ One longitudinal study found initial increased involvement in LGB+-related activities was associated with increased alcohol and marijuana use among LGB+ youth, but substance use eventually decreased over time.^37^ Therefore, once settled in their sexual identities, LGB+ youth may feel less compelled to socially smoke.

It is also possible that youth and young adults smoke as a coping mechanism for the stress associated with coming out.^20^ Psychological distress, discrimination, and mental health disorders are consistently found to be correlated with cigarette smoking in the literature.^39,40,41,42^ LGB+ young people exhibit greater incidence of poor mental health and stress,^20^ and nationally representative data show bisexual populations have higher prevalence of severe psychological distress than other LG+ populations.^43^ Publicly identifying as LGB+ makes individuals vulnerable to social stressors in the form of stigma, prejudice, and discrimination, a phenomenon called *minority stress*.^20^ Recently out LGB+ individuals may smoke as a reaction to newly experienced discrimination.^38,44^ A study by Newcomb et al^4^ found psychological distress and LGB+ violence and discrimination predicted cigarette smoking and higher rates of smoking among LGB+ youth.^4^ However, other research suggests individuals who privately but not publicly identify as LGB+ can experience stress caused by internalized homonegativity^12,45^; in this case, coming out may actually alleviate internalized stress. More research is needed to better understand the role of minority stress and LGB+ mental health on risk of smoking.

### Limitations

There are limitations of our study. First, we do not know participants’ sexual identity before entering and after leaving the study. Second, participants’ reported sexual identity may not align with their public sexual identity. It is possible some participants privately identified as LGB+ during data collection but publicly identified as heterosexual. Further, sexual orientation is comprised of 3 domains (identity, attraction, behavior), and we only address identity in this analysis. It is difficult to make conclusions on mechanisms without more data on environmental and interpersonal contexts. Third, although we group together participants with the same identity patterns, the LGB+ community is diverse, and individuals within the same identity group may differ on factors related to smoking risk, including familial support and social environments. Participants across groups may also differ on unmeasured confounders, and we cannot rule out residual confounding. Fourth, some identity categories had small sample sizes, leading to imprecise estimates with wide confidence intervals. Larger samples are needed to explore granularities in sexual identity and smoking, including risks associated with gender identity transitions for transgender and nonbinary youth and intersectional identities (eg, combinations of sexual identity, race/ethnicity, gender, etc). Fifth, our assumption of data being missing at random for multiple imputation may not be met if there are unmeasured variables associated with missing data.

## Conclusions

Our study represents one of the first analyses to explore the nuances of sexual identity development and timing associated with cigarette smoking initiation on a national scale, to our knowledge. Our findings stress the importance of recognizing that LGB+ young people in different stages of identity development and with different LGB+ identities have different risks of smoking. Those who have recently changed identities may be more at risk of smoking initiation than those who have identified as LGB+ consistently for some time. Our data suggest young people who change to or from a bisexual identity are particularly susceptible. Future research should address mechanisms underlying the association between changes in sexual identity and cigarette smoking to inform tailored smoking prevention programs and tobacco regulations.

## Notes

Supplement. eFigure. Flowchart of Exclusion/Inclusion Criteria eTable 1. Descriptive Baseline Characteristics Stratified by Eight-Level Sexual Identity Categories Among 7,843 Never Cigarette Smokers, Population Assessment of Tobacco and Health Study, 2013-2018 eTable 2. Prevalence of sexual identity patterns across four waves of data among 350 participants who change sexual identity over follow-up, but are not classified as coming out LGB+, Population Assessment of Tobacco and Health Study, 2013-2018 eTable 3. Descriptive baseline characteristics among never cigarette smokers retained and lost to follow-up, Population Assessment of Tobacco and Health Study, 2013-2018 eTable 4. Sex-Stratified Cigarette Smoking at Wave 4 by Four-Level Sexual Identity Categories among 7,843 Never Cigarette Smokers at Wave 1, Population Assessment of Tobacco and Health Study, 2013-2018 eTable 5. Age-Stratified Cigarette Smoking at Wave 4 by Four-Level Sexual Identity Categories among 7,843 Never Cigarette Smokers at Wave 1, Population Assessment of Tobacco and Health Study, 2013-2018
