# Supplementary material for: Association of Coming Out as Lesbian, Gay, and Bisexual+ and Risk of Cigarette Smoking in a Nationally Representative Sample of Youth and Young Adults
Source: JAMA Pediatr. 2020 Oct 26;175(1):1–9. doi: 10.1001/jamapediatrics.2020.3565 (PMC7589064; doi:10.1001/jamapediatrics.2020.3565)
Supplement: Supplement. — eFigure. Flowchart of Exclusion/Inclusion Criteria eTable 1. Descriptive Baseline Characteristics Stratified by Eight-Level Sexual Identity Categories Among 7,843 Never Cigarette Smokers, Population Assessment of Tobacco and Health Study, 2013-2018 eTable 2. Prevalence of sexual identity patterns across four waves of data among 350 participants who change sexual identity over follow-up, but are not classified as coming out LGB+, Population Assessment of Tobacco and Health Study, 2013-2018 eTable 3. Descriptive baseline characteristics among never cigarette smokers retained and lost to follow-up, Population Assessment of Tobacco and Health Study, 2013-2018 eTable 4. Sex-Stratified Cigarette Smoking at Wave 4 by Four-Level Sexual Identity Categories among 7,843 Never Cigarette Smokers at Wave 1, Population Assessment of Tobacco and Health Study, 2013-2018 eTable 5. Age-Stratified Cigarette Smoking at Wave 4 by Four-Level Sexual Identity Categories among 7,843 Never Cigarette Smokers at Wave 1, Population Assessment of Tobacco and Health Study, 2013-2018 [file jamapediatr-e203565-s001.pdf]

Supplemental Online Content

Harlow AF, Lundberg D, Raifman JR, et al. Association of coming out as lesbian, gay, and bisexual+ and risk of cigarette smoking in a nationally representative sample of youth and young adults. *JAMA Pediatr*. Published online October 26, 2020.
doi:10.1001/jamapediatrics.2020.3565

eFigure. Flowchart of Exclusion/Inclusion Criteria
eTable 1. Descriptive Baseline Characteristics Stratified by Eight-Level Sexual Identity Categories Among 7,843 Never Cigarette Smokers, Population Assessment of Tobacco and Health Study, 2013-2018
eTable 2. Prevalence of sexual identity patterns across four waves of data among 350 participants who change sexual identity over follow-up, but are not classified as coming out LGB+, Population Assessment of Tobacco and Health Study, 2013-2018
eTable 3. Descriptive baseline characteristics among never cigarette smokers retained and lost to follow-up, Population Assessment of Tobacco and Health Study, 2013-2018
eTable 4. Sex-Stratified Cigarette Smoking at Wave 4 by Four-Level Sexual Identity Categories among 7,843 Never Cigarette Smokers at Wave 1, Population Assessment of Tobacco and Health Study, 2013-2018
eTable 5. Age-Stratified Cigarette Smoking at Wave 4 by Four-Level Sexual Identity Categories among 7,843 Never Cigarette Smokers at Wave 1, Population Assessment of Tobacco and Health Study, 2013-2018

This supplemental material has been provided by the authors to give readers additional information about their work.

eTable 1. Descriptive Baseline Characteristics Stratified by Eight-Level Sexual Identity Categories Among 7,843 Never Cigarette Smokers, Population Assessment of Tobacco and Health Study, 2013-2018

Wave 1 characteristic[a]	Consistently Heterosexual (n=6,707)	Consistently LG+ (n=100)	Consistently Bisexual (n=85)	Consistently LGB+ with change to/from Bisexual (n=99)	Coming out Bisexual (n=339)	Coming out LG+ (n=163)	Other LGB+ Patterns with change to/from Bisexual (n=189)	Other LG+ Patterns (n=161)
% of total sample	87.1	1.3	1.1	1.2	3.2	1.78	2.1	87.1
Age, mean (SE)	20.1 (0.75)	20.7 (5.8)	20.4 (6.2)	19 (6.5)	17.5 (2.7)	18.9 (6.1)	19.8 (5.5)	20.8 (5.2)
Female sex	50.7	59.8	61.9	71.5	65.6	57	69.7	50.7
Race								
NH White	52.9	53.1	55.1	52.3	57.0	47.1	37.1	41.0
NH Black	14.9	23.2	17.5	9.0	12.9	14.1	17.6	21.5
Hispanic	20.3	18.2	16.1	14.0	16.7	20.7	32.5	28.7
Asian or Other	11.8	5.5	11.4	2.7	13.3	18.0	12.8	8.9
Education[b]								
< HS	11.0	10.9	7.0	19.5	10.9	14.8	23.3	
HS Grad	24.6	23.7	24.0	19.3	24.9	24.2	22.7	
>HS	64.5	65.5	69.0	61.3	64.2	61.0	54.1	
US census region								
Northeast	17.8	27.5	12.8	10.1	12.6	12.0	17.3	19.1
Midwest	21.1	21.7	31.9	18.9	25.0	36.2	20.1	22.1
South	36.2	32.0	17.5	49.2	38.8	27.6	40.4	36.3
West	24.9	18.8	37.8	21.7	23.6	24.2	22.2	22.6
Urban residing	95.5	87.8	98.1	94.4	96.5	93.7	98.7	95.5

Abbreviations: LGB+, Lesbian, Gay, Bisexual and something else besides heterosexual; LG+, Lesbian, Gay, and something else besides heterosexual; SE, standard error; NH, Non-Hispanic
[a]Descriptive statistics calculated using sample-weighted percentages and sample-weighted means
[b]For participants <18 years old, parental education is reported.

© 2020 Harlow AF et al. *JAMA Pediatrics.*

eTable 2. Prevalence of sexual identity patterns across four waves of data among 350 participants who change sexual identity over follow-up, but are not classified as coming out LGB+, Population Assessment of Tobacco and Health Study, 2013-2018				
W1 LGB+[a]	W2 LGB+[a]	W3 LGB+[a]	W4 LGB+[a]	Weighted %
0	1	1	0	6.5
0	1	0	0	14.5
0	0	1	0	16.5
1	0	0	0	29.3
1	0	0	1	1.7
1	0	1	1	2.9
1	1	1	0	9.5
1	0	1	0	6.0
1	1	0	1	4.5
1	1	0	0	8.6

Abbreviations: W1, wave 1; W2, wave 2; W3, wave 3; W4, wave 4; LGB+, Lesbian, Gay, Bisexual and something else besides heterosexual

[a] Indicates LGB+ status at each wave. 1=Identifies as LGB+ at specified wave, 0= Identifies as heterosexual at specified wave

© 2020 Harlow AF et al. *JAMA Pediatrics.*

**eTable 3. Descriptive baseline characteristics among never cigarette smokers retained and lost to follow-up, Population Assessment of Tobacco and Health Study, 2013-2018**		
Wave 1 characteristic[a]	Retained (n=7,843)	Lost to Follow-up (n=3,197)[b]
Age, mean (SE)	19.9 (0.7)	20.4 (0.9)
Female sex	52.3	51.8
Race		
NH White	52.4	52.2
NH Black	15.1	20.4
Hispanic	20.5	13.4
Asian or Other	12.0	14.0
Education[c]		
< High School	11.7	12.2
Graduated High School	24.5	26.8
>High School	63.8	61.0
US census region		
Northeast	17.6	19.8
Midwest	21.6	19.6
South	36.1	36.4
West	24.8	24.0
Urban residing	95.6	95.1
Sexual Identity		
LG	1.1	1.3
Heterosexual	93.7	94.4
Bisexual	3.0	2.6
Other non-heterosexual identity	2.2	1.9

Abbreviations: NH, Non-Hispanic; LGB+, Lesbian, Gay, Bisexual and something else besides heterosexual;
[a]Descriptive statistics calculated using sample-weighted percentages and sample-weighted means
[b]Never cigarette smokers at baseline who did not complete all four waves of data
[c]For participants <18 years old, parental education is reported.

© 2020 Harlow AF et al. *JAMA Pediatrics.*

eTable 4. Sex-Stratified Cigarette Smoking at Wave 4 by Four-Level Sexual Identity Categories among 7,843 Never Cigarette Smokers at Wave 1, Population Assessment of Tobacco and Health Study, 2013-2018

Sexual Identity Category	Cigarette use (%)[a]	Odds Ratio (95% CI)	Adjusted Odds Ratio (95% CI)[b]	Predicted Probabilities (95% CI) [b,c]
**Males (n=3,777)**				
**Smoking Initiation by Wave 4[d]**				
Total	16.6	--	--	--
Consistently Heterosexual	16.4	1.0 (REF)	1.0 (REF)	0.202 (0.175-0.229)
Consistently LGB+	14.9	0.83 (0.43-1.58)	0.90 (0.47 to 1.71)	0.186 (0.088-0.283)
Coming out LGB+	23.4	1.59 (1.08-2.33)	1.30 (0.89 to 1.91)	0.248 (0.181-0.315)
Other LGB+ Patterns	15.5	0.98 (0.47-2.01)	1.08 (0.56 to 2.06)	0.214 (0.105-0.324)
**Current Smoker at Wave 4[e]**				
Total	7.5	--	--	--
Consistently Heterosexual	7.3	1.0 (REF)	1.0 (REF)	0.088 (0.072-0.104)
Consistently LGB+	6.3	0.96 (0.32-2.85)	0.96 (0.32-2.85)	0.085 (0.003-0.167)
Coming out LGB+	11.6	1.39 (0.86-2.24)	1.39 (0.86-2.24)	0.118 (0.064-0.173)
Other LGB+ Patterns	11.0	1.67 (0.72-3.89)	1.90 (0.84-4.31)	0.155 (0.040-0.270)
**Females (n=4,066)**				
**Smoking Initiation by Wave 4[d]**				
Total	11.8	--	--	--
Consistently Heterosexual	10.3	1.0 (REF)	1.0 (REF)	0.126 (0.102-0.150)
Consistently LGB+	18.1	2.00 (1.26-3.18)	1.94 (1.23-3.00)	0.090 (0.026-0.153)
Coming out LGB+	22.7	2.61 (1.84-3.68)	2.14 (1.57-2.93)	0.195 (0.120-0.271)
Other LGB+ Patterns	18.3	1.97 (1.37-2.85)	1.93 (1.36-2.73)	0.190 (0.073-0.307)
**Current Smoker at Wave 4[e]**				
Total	5.2	--	--	--
Consistently Heterosexual	4.4	1.0 (REF)	1.0 (REF)	0.112 (0.095-0.129)
Consistently LGB+	9.5	2.32 (1.24-4.34)	2.22 (1.22-4.04)	0.196 (0.126-0.267)
Coming out LGB+	11.4	2.80 (1.84-4.26)	2.24 (1.48-3.40)	0.213 (0.160-0.266)
Other LGB+ Patterns	7.1	1.66 (1.02-2.72)	1.54 (0.96-2.49)	0.196 (0.141-0.251)

Abbreviations: LGB+, Lesbian, Gay, Bisexual and something else besides heterosexual; CI, confidence interval

[a]Sample-weighted percentages

[b]Regression models and predicted probabilities were sample weighted and adjusted for urban residence, sex, race/ethnicity, education, census region

[c]Predicted probabilities calculated at the mean of model covariates

[d]Reported ever smoking by wave 4

[e]Defined as past 30-day smoking at wave 4

Biological sex interaction term p-values: smoking initiation=0.04; current smoking=0.31

© 2020 Harlow AF et al. *JAMA Pediatrics.*

eTable 5. Age-Stratified Cigarette Smoking at Wave 4 by Four-Level Sexual Identity Categories among 7,843 Never Cigarette Smokers at Wave 1, Population Assessment of Tobacco and Health Study, 2013-2018

Identity Trajectory	Cigarette use (%)	Odds Ratio (95% CI)	Adjusted Odds Ratio (95% CI)[a]	Predicted Probabilities (95% CI) [ab]
**<18 years old (n=5,343)**				
**Ever Cigarette Use**				
Total	19.9			--
Consistently Heterosexual	18.7	1.0 (REF)	1.0 (REF)	0.161 (0.146-0.177)
Consistently LGB+	26.2	1.60 (1.07-2.40)	1.75 (1.14-2.71)	0.252 (0.168-0.337)
Coming out LGB+	26.6	1.59 (1.19-2.12)	1.71 (1.28-2.27)	0.247 (0.196-0.297)
Other LGB+ Patterns	26.3	1.59 (1.23-2.25)	1.77 (1.25-2.49)	0.253 (0.188-0.318)
**Current (Past 30-day) Cigarette Use**				
Total	9.9			--
Consistently Heterosexual	9.3	1.0 (REF)	1.0 (REF)	0.081 (0.069-0.092)
Consistently LGB+	13.4	1.56 (0.91-2.7)	1.80 (1.00-3.11)	0.137 (0.069-0.205)
Coming out LGB+	13.7	1.54 (1.16-2.04)	1.69 (1.25-2.22)	0.129 (0.098-0.161)
Other LGB+ Patterns	11.8	1.35 (0.86-2.13)	1.43 (0.96-2.39)	0.119 (0.071-0.167)
**≥18 years old (n=2,500)**				
**Ever Cigarette Use**				
Total	10.6			--
Consistently Heterosexual	10.2	1.0 (REF)	1.0 (REF)	0.108 (0.086-0.130)
Consistently LGB+	11.7	1.14 (0.64-2.06)	1.22 (0.68-2.17)	0.128 (0.063-0.194)
Coming out LGB+	17.6	1.95 (1.04-3.66)	2.08 (1.08-4.01)	0.201 (0.098-0.304)
Other LGB+ Patterns	11.6	1.17 (0.61-2.25)	1.05 (0.53-2.10)	0.113 (0.045-0.181)
**Current (Past 30-day) Cigarette Use**				
Total	4.1			--
Consistently Heterosexual	3.8	1.0 (REF)	1.0 (REF)	0.039 (0.025-0.053)
Consistently LGB+	5.4	1.45 (0.52-4.08)	1.52 (0.56-4.12)	0.058 (0.002-0.114)
Coming out LGB+	8.0	2.31 (0.91-5.85)	2.61 (1.00-6.82)	0.096 (0.019-0.173)
Other LGB+ Patterns	6.4	1.76 (0.76-4.10)	1.45 (0.57-3.65)	0.056 (0.008-0.103)

Abbreviations: LGB+, Lesbian, Gay, Bisexual and something else besides heterosexual; CI, confidence interval
[a]Sample-weighted percentages
[b]Regression models and predicted probabilities were sample weighted and adjusted for urban residence, sex, race/ethnicity, education, census region
[c]Predicted probabilities calculated at the mean of model covariates
[d]Reported ever smoking by wave 4
[e]Defined as past 30-day smoking at wave 4
Age interaction term p-values: smoking initiation=0.66; current smoking=0.87

© 2020 Harlow AF et al. *JAMA Pediatrics.*

eFigure. Flowchart of Exclusion/Inclusion Criteria

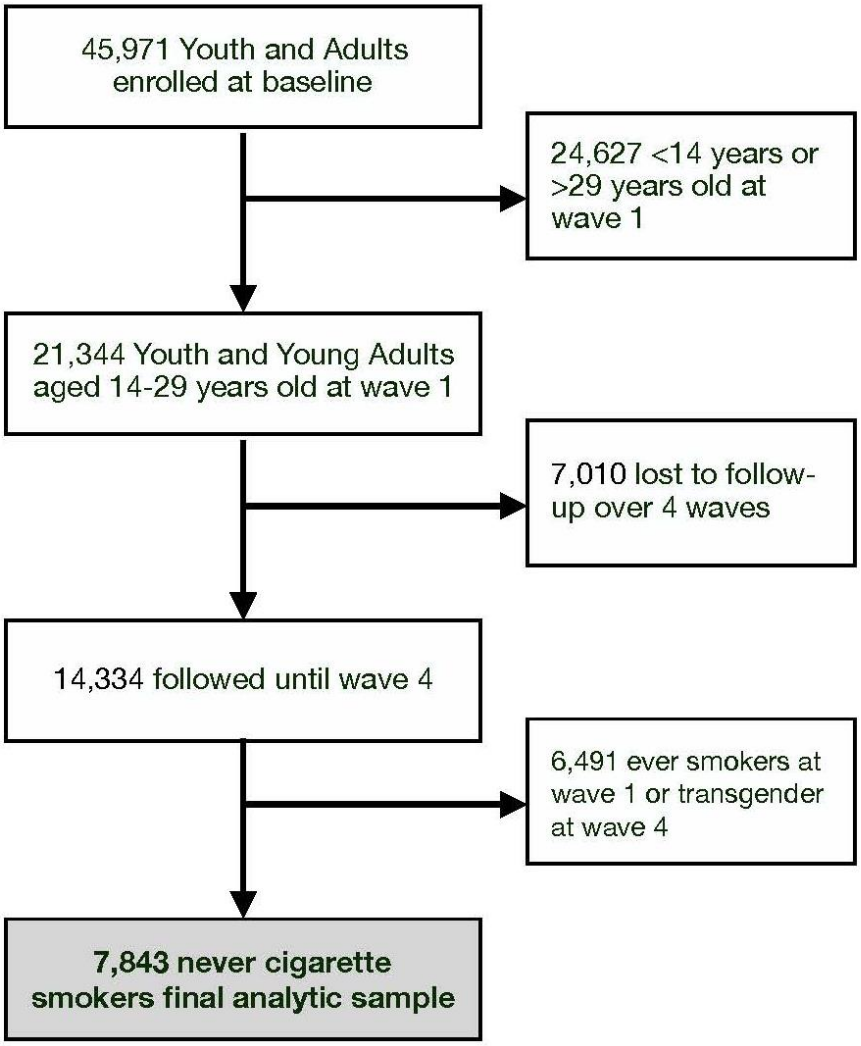

© 2020 Harlow AF et al. *JAMA Pediatrics.*
